# Characterizing neck injuries in the national football league: a descriptive epidemiology study

**DOI:** 10.1186/s12891-023-06830-y

**Published:** 2023-09-02

**Authors:** Bobby Dow, Dakota Doucet, Sree M. Vemu, Venkat Boddapati, Rex A. W. Marco, Takashi Hirase

**Affiliations:** 1 Houston Methodist Orthopedics and Sports Medicine, 6445 Main Street, Suite 2500, Houston, TX 77030 USA; 2 Texas A&M University Health Science Center College of Medicine, 8447 Riverside Pkwy, Bryan, TX 77807 USA; 3 Department of Orthopaedic Surgery, Hospital for Special Surgery, 535 E. 70th St, New York, NY 10021 USA

**Keywords:** Tackling techniques, American football, Cervical spine injury, Neck injury, Sports trauma

## Abstract

**Background:**

Neck injury is a common and often debilitating injury among athletes participating in American football. Limited data exists regarding neck injuries among elite athletes in the National Football League (NFL). To characterize the epidemiology of non-season ending, season-ending, and career-ending neck injuries in the NFL from 2016 through 2021.

**Methods:**

Athletes who sustained neck injuries were identified using the NFL’s injured reserve (IR) list between the 2016 and 2021 seasons. Demographics and return to sport (RTS) data were collected. Available game footages were reviewed to identify the mechanism of injury (MOI). Injury incidence rates were calculated based on per team play basis.

**Results:**

During the 6-year study period, 464 players (mean age 26.8 ± 3.2 years) were placed on the injury reserve list due to neck injuries. There were 285 defensive players and 179 offensive players injured (61.4 vs 38.6%, respectively, *p* < 0.001). Defensive back was the most common position to sustain a neck injury (111 players, 23.9%). 407 players (87.7%) sustained non-season-ending injuries with a mean RTS at 9.2 ± 11.3 days. 36 players (7.8%) sustained season-ending injuries with a mean RTS at 378.6 ± 162.0 days. 21 players (4.5%) sustained career-ending injuries. The overall incidence of neck injuries was 23.5 per 10,000 team plays. The incidence of season-ending injuries and career-ending injuries were 1.82 and 1.06 per 10,000 team plays, respectively. There were 38 injuries with available footages for MOI assessment (23 non-season-ending, 9 season-ending, 6 career-ending). Head-to-head contact was seen in 15 injuries (39.5%), head-down tackling in 11 injuries (28.9%), direct extremity-to-head contact in 7 injuries (18.4%), and head-to-ground contact in 5 injuries (13.2%). There was no significant difference in age, position, or MOI among players sustaining non-season-ending, season-ending, and career-ending injuries.

**Conclusion:**

There is a high incidence of neck injuries among NFL athletes with predictable MOIs including head-to-head contact, head-down tackling, direct extremity-to-head contact, and head-to-ground contact. Defensive players were more likely to sustain neck injuries compared to offensive players. Defensive back was the most common position to sustain a neck injury.

**Level of evidence:**

III.

## Introduction

Neck injury is a common and often debilitating injury among athletes participating in American football [1–3]. These injuries have been associated with poor techniques including head-down tackling, head-across the bow tackling, and spear tackling [4–6]. Spear tackling is a form of head-down tackling technique utilizing the crown of the helmet that involves the tackler placing the head and neck in a neutral or flexed position during a tackle where axial compression forces are transferred directly into the cervical spine. This technique has been associated with generating catastrophic vertebral body failure and quadriplegia [6]. Since the prohibition of spear tackling in 1976, the rate of catastrophic spine injuries has decreased significantly among all age groups [6–9]. Although incidence decreased, catastrophic brain and spinal cord injuries remain prevalent in American Football particularly during competition versus practice and in part due to the increasing protection received from modern helmets [5, 7]. In the National Football League (NFL), information regarding each player’s injuries and detailed statistics are available via public domains. In the past several years, there have been multiple publications using this publicly available data that has suggested injuries experienced by NFL players to be unique in nature in terms of mechanisms of injury and timeline to return to sports [10, 11]. However, limited data exists regarding neck injuries among elite athletes in the NFL, and to our knowledge, no recent studies have characterized the neck injuries in the NFL. Thus, the primary objective of this study was to characterize the epidemiology of non-season ending, season-ending, and career-ending neck injuries in the NFL from 2016 through 2021. Secondary objectives were (1) to examine the variation in time to return-to-sport (RTS) after neck injuries and (2) to characterize the mechanism of injury (MOI) that leads to neck injuries in the NFL.

## Materials and methods

### Study population

The study protocol was exempt from the local institutional review board due to the publicly available nature of the data for analysis. Athletes who sustained neck injuries were identified using the NFL’s injured reserve (IR) list between the 2016 and 2021 seasons. This data is publicly available on NFL.com [12]. Exclusion criteria were injuries not identified as neck injury in the IR list. Players with neck injuries and other concurrent injuries were counted.

### Data collection

Demographics and return to sport (RTS) data were collected. Demographic variables including a player’s age, position, date of injury, and date of return to sport were recorded. Players were categorized by their positions as well as their role as an offensive or defensive player. A player was deemed to have RTS if he played in a single NFL game after the injury as previously defined [10, 11]. Players with multiple injuries per season were counted as separate entries if the player played in a game in between injuries. Available slow-motion game footages were reviewed to identify the mechanism of injury (MOI). A previously described method was utilized for tackle analysis if a tackle was related to the MOI [6].

Injury incidence rates were calculated based on per team play basis. The number of team plays were determined by the NFL and included all team plays of all 32 teams during the 2016 through 2021 seasons [13]. A single exposure, or one team play was defined as one team participating in one offensive, defensive, or special teams play.

### Statistical analysis

Data analysis was performed using SPSS statistical software (Version 25.0; SPSS, Inc, Chicago IL). The Chi-Square or Fisher’s exact test was used to analyze categorical data and the two-tailed student *t-*test or the Mann–Whitney U test was used to analyze continuous data. A *p*-value < 0.05 was considered statistically significant.

Tables 3 and 4 are listed as rate per 100,000 whereas Tables 1 and 2 are listed as rate per 10,000.

**Table 1:** Overall Neck Injuries From 2016 Through 2021

	**Position**	**N**	**Mean age ± SD (years)**	**Incidence per 10,000 Team Plays (95% CI)**
**Defense**	LB	77	25.8 ± 3.2	3.9 (3.1–4.9)
	DT	37	27.6 ± 3.5	1.9 (1.3–2.6)
	DE	60	26.9 ± 3.4	3.0 (2.3–3.9)
	DB	111	26.8 ± 3.2	5.6 (4.6–6.7)
**Offense**	QB	9	26.7 ± 4.2	0.5 (0.2–0.8)
	RB	36	25.8 ± 2.8	1.8 (1.3–2.5)
	WR	31	26.0 ± 2.5	1.6 (1.1–2.2)
	OG	34	28.4 ± 3.4	1.7 (1.2–2.4)
	OT	22	28.0 ± 2.6	1.1 (0.7–1.7)
	TE	34	26.1 ± 2.3	1.7 (1.2–2.4)
	C	13	28.5 ± 3.3	0.7 (0.4–1.1)
**Overall**		464	26.8 ± 3.2	23.5 (21.4–25.7)

*SD* Standard deviation, *CI* Confidence interval, *LB* Linebacker, *DT* Defensive tackle, *DE* Defensive end, *DB* Defensive back, *QB* Quarterback, *RB* Running back, *WR* Wide receiver, *OG* Offensive guard, *OT* Offensive tackle, *TE* Tight end, *C* Center

**Table 2:** Non-Season-Ending Neck Injuries From 2016 Through 2021

	**Position**	**N**	**Mean age ± SD (years)**	**RTS ± SD (days)**	**Incidence per 10,000 Team Plays (95% CI)**
**Defense**	LB	67	25.8 ± 3.3	8.8 ± 7.2	3.4 (2.7–4.3)
	DT	33	27.8 ± 3.5	9.3 ± 19.7	1.7 (1.2–2.3)
	DE	52	26.9 ± 3.4	9.3 ± 9.8	2.6 (2.0–3.4)
	DB	102	26.9 ± 3.2	9.9 ± 8.8	5.2 (4.2–6.2)
**Offense**	QB	6	26.3 ± 4.5	3.0 ± 2.0	0.3 (0.1–0.6)
	RB	26	25.0 ± 3.0	9.0 ± 7.4	1.3 (0.9–1.9)
	WR	25	25.8 ± 2.6	5.7 ± 3.8	1.3 (0.8–1.8)
	OG	32	28.3 ± 3.4	7.9 ± 7.2	1.6 (1.6–1.7)
	OT	20	28.0 ± 2.8	10.1 ± 11.5	1.0 (0.6–1.5)
	TE	32	25.8 ± 2.4	8.7 ± 13.6	1.6 (1.6–1.7)
	C	12	28.0 ± 3.3	28.0 ± 30.2	0.6 (0.3–1.0)
**Overall**		407	26.7 ± 3.3	9.2 ± 11.3	20.6 (18.7–22.7)

*SD* Standard deviation, *RTS* Return to sport, *CI* Confidence interval, *LB* Linebacker, *DT* Defensive tackle, *DE* Defensive end, *DB* Defensive back, *QB* Quarterback, *RB* Running back, *WR* Wide receiver, *OG* Offensive guard, *OT* Offensive tackle, *TE* Tight end, *C* Center

## Results

During the 6-year study period, 464 players (mean age 26.8 ± 3.2 years) were placed on the IR list due to neck injuries (Table 1). There were 285 defensive players and 179 offensive players injured (61.4 vs 38.6%, respectively, *p* < 0.001). Defensive back was the most common position to sustain a neck injury (111 players, 23.9%). Of these, 407 players (87.7%) sustained non-season-ending injuries with a mean RTS at 9.2 ± 11.3 days (Table 2). There were 36 players (7.8%) who sustained season-ending injuries with a mean RTS at 378.6 ± 162.0 days (Table 3). There were 21 players (4.5%) who sustained career-ending injuries (Table 4). The overall incidence of neck injuries was 23.5 per 10,000 team plays. The incidence of season-ending injuries and career-ending injuries were 1.82 and 1.06 per 10,000 team plays, respectively.

**Table 3:** Season-Ending Neck Injuries From 2016 Through 2021

	**Position**	**N**	**Mean age ± SD (years)**	**RTS ± SD (days)**	**Incidence per 100,000 Team Plays (95% CI)**
**Defense**	LB	5	25.3 ± 2.9	365.5 ± 79.8	2.5 (0.9–5.6)
	DT	2	25.5	352.5	1.0 (0.2–3.3)
	DE	7	25.7 ± 2.3	377.7 ± 138.1	3.5 (1.5–7.0)
	DB	5	23.0 ± 1.3	348.0 ± 84.4	2.5 (0.9–5.6)
**Offense**	QB	2	29.0	87.0	1.0 (0.2–3.3)
	RB	7	26.7 ± 3.5	405.5 ± 166.7	3.5 (1.5–7.0)
	WR	4	27.3 ± 4.0	320.0 ± 56.7	2.0 (0.6–4.9)
	OG	1	32.0	259.0	0.5 (0.0–2.5)
	OT	1	29.0	426.0	0.5 (0.0–2.5)
	TE	2	25.0	613.5	1.0 (0.2–3.3)
	C	0	N/A	N/A	0.0 (0.0–0.0)
**Overall**		36	26.3 ± 3.5	364.2 ± 152.1	18.2 (13.0–25.0)

*SD* Standard deviation, *RTS* Return to sport, *CI* Confidence interval, *LB* Linebacker, *DT* Defensive tackle, *DE* Defensive end, *DB* Defensive back, *QB* Quarterback, *RB* Running back, *WR* Wide receiver, *OG* Offensive guard, *OT* Offensive tackle, *TE* Tight end, *C* Center

**Table 4:** Career-Ending Neck Injuries From 2016 Through 2021

	**Position**	**N**	**Mean age ± SD (years)**	**Incidence per 100,000 Team Plays (95% CI)**
**Defense**	LB	5	26.2	2.5 (0.9–5.6)
	DT	2	25.5	1.0 (0.2–3.3)
	DE	1	31.0	0.5 (0.0–2.5)
	DB	4	28.5	2.0 (0.6–4.9)
**Offense**	QB	1	24.0	0.5 (0.0–2.5)
	RB	3	31.3	1.5 (0.4–4.1)
	WR	2	26.5	1.0 (0.2–3.3)
	OG	1	31.0	0.5 (0.0–2.5)
	OT	1	26.0	0.5 (0.0–2.5)
	TE	0	N/A	0.0 (0.0–0.0)
	C	1	34.0	0.5 (0.0–2.5)
**Overall**		21	26.3 ± 3.5	10.6 (6.8–16.0)

*SD* Standard deviation, *RTS* Return to sport, *CI* Confidence interval, *LB* Linebacker, *DT* Defensive tackle, *DE* Defensive end, *DB* Defensive back, *QB* Quarterback, *RB* Running back, *WR* Wide receiver, *OG* Offensive guard, *OT* Offensive tackle, *TE* Tight end, *C* Center

**Fig. 1:**
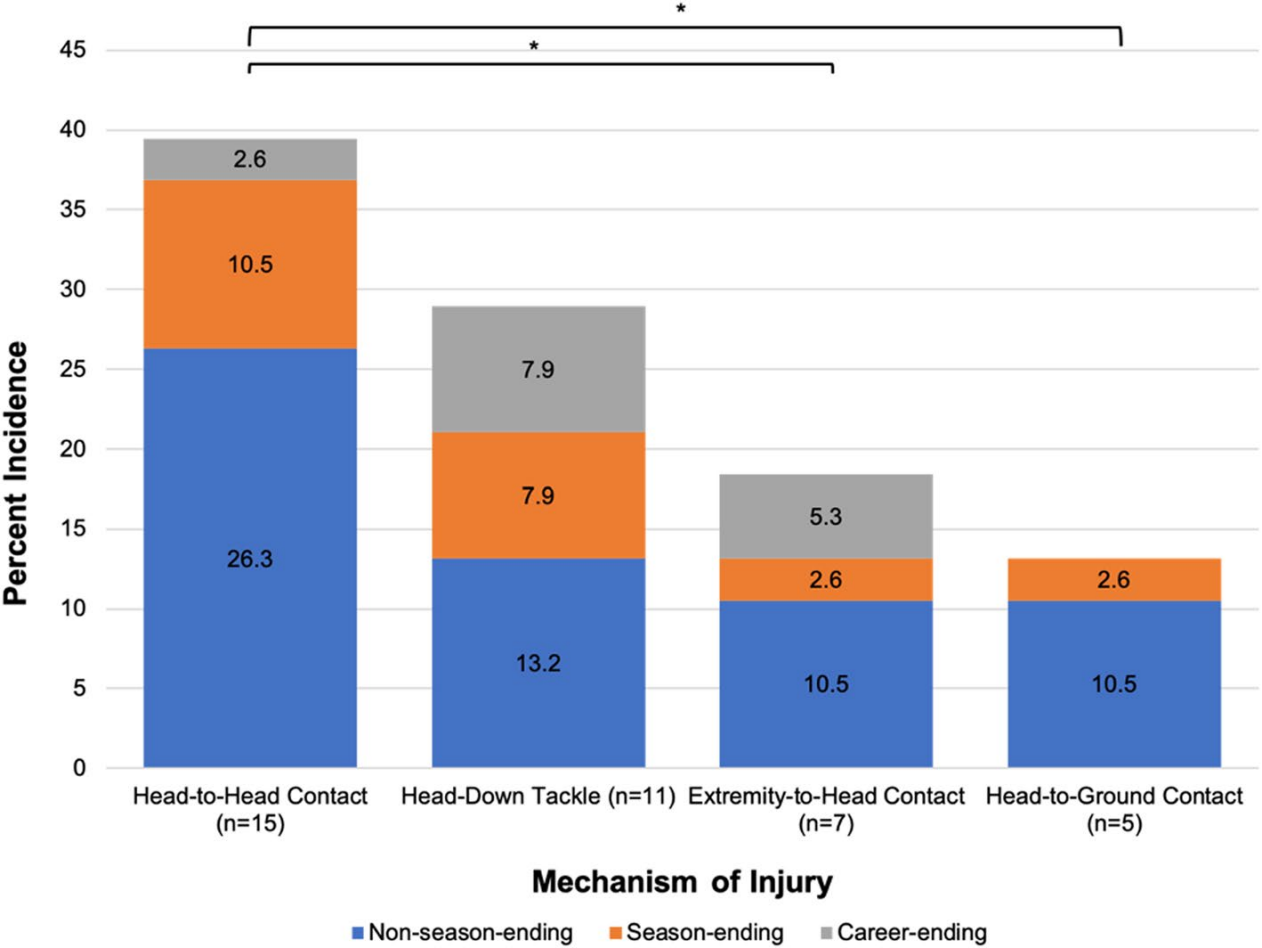
Mechanism of Injury Assessment of Available Footages. *p < 0.05; Statistically significant values. Abbreviations: NFL = National Football League; IR = injury reserve; RTS = return-to-sport; MOI = mechanism of injury; SD = standard deviation; RTS = return to sport; CI = confidence interval; LB = linebacker; DT = defensive tackle; DE = defensive end; DB = defensive back; QB = quarterback; RB = running back; WR = wide receiver; OG = offensive guard; OT = offensive tackle; TE = tight end; C = center

There were 38 injuries with available footage for MOI assessment (23 non-season-ending, 9 season-ending, 6 career-ending) (Fig. 1). Head-to-head contact was seen in 15 injuries (39.5%), head-down tackling in 11 injuries (28.9%), direct extremity-to-head contact in 7 injuries (18.4%), and head-to-ground contact in 5 injuries (13.2%). Head-down tackling was associated with the highest incidence of season-ending injuries (7.9%), although not statistically significant. There was no significant difference in age, position, or MOI among players sustaining non-season-ending, season-ending, and career-ending injuries (*p* > 0.05).

## Discussion

The principal finding of this study demonstrates that there is a high incidence of neck injuries among NFL athletes with predictable MOIs, including head-to-head contact, head-down tackling, direct extremity-to-head contact, and head-to-ground contact. Defensive players were more likely to sustain neck injuries compared to offensive players. The defensive back was the most common position to sustain a neck injury.

Our findings were similar to results from prior studies utilizing older data within the NFL. A prior systematic review of 147 articles by Kluczynski et al. showed spine injuries to account for 7% of all NFL orthopedic injuries [14]. Beaulier-Jones et al. evaluated 2203 NFL combine athletes from 2009 and 2015 and found cervical spine injuries in 384 players, with defensive players having a significantly higher likelihood of injury than all other positions [15]. Similarly, our six-year time frame had 464 total players with neck injuries, and defensive players were over 60% of the population. This finding may partially be attributed to a smaller space available for the subaxial spinal cord among defensive players. A study by Presciutti et al. evaluated 103 male athletes participating in the 2005 and 2006 NFL Scouting Combine and found defensive players to have significantly reduced mean subaxial cervical space available for the cord compared to other positional players [16]. The authors speculated that this may largely be secondary to repeated cervical spine trauma from blocking or tackling. Furthermore, a retrospective NFL injury database study by Mall et al. investigating axial skeleton injuries from 2000–2010 found that cervical spine injuries are ten times more likely to occur in games when defensive players are blocking or tackling [17].

There were four major MOIs identified in our study that lead to neck injuries: direct head-to-head contact, head-down tackling, extremity-to-head contact, and head-to-ground contact. Although not statistically significant, our study found that head-down tackling was associated with the highest probability of serious injuries with more than 50% of the injuries being associated with head-down tackling leading to season-ending or career ending injuries. This was consistent with prior studies that have also identified that head-down tackling is the most dangerous tackling method with the highest risk for devastating injuries including death and paralysis [5]. Other than head-down tackling, which always occurred during defense, injuries from direct head-to-head contact, extremity-to-head contact, and head-to-ground contact equally occurred in both offense and defense. Although prior studies have identified at-risk tackling techniques, our findings also suggest that neck injuries leading to placement onto the IR list also occurs with non-tackle-related incidents with direct contact to the head. However, this finding is in contrast to prior reports indicating that although direct head impact commonly leads to cranial injuries and concussions, it rarely leads to cervical spine injuries [18]. This has been attributed to the remarkable flexibility of the neck that serves as a protective factor during a direct head impact [18]. However, other studies have shown that increased constraints to the motion of the cervical spine during the head impact may lead to higher likelihood of injury [19]. This is consistent with the results in our study, which demonstrated that all direct head impacts leading to neck injuries were associated with secondary constraints to motion. This consisted of the head pocketing into either the ground or other surrounding players that prevented the ability of the head and neck to escape point of impact. These results suggest that to prevent neck injuries, not only should adequate attention be placed towards proper tackling methods, but also towards appropriate techniques for avoiding direct head impacts.

There were several limitations to this study. Although the data was obtained directly from the league’s official website, the acquired data remains susceptible to variability in reporting. Furthermore, the NFL reports generalized “neck injury” within their IR list; however, does not specify the type of injury. The NFL also reports all injuries in which the player was placed on the injured reserve and therefore leads to a misclassification bias when calculating RTS date. Given how injuries are reported, it is difficult to determine if players with neck injuries and other injuries, whether the neck injury or the other injury is keeping the player from returning to NFL play. Further prospective studies that include the diagnosis, imaging, and examination is necessary to further determine the incidence and consequences of specific neck-related injuries. For season ending injuries, players may have healed over the off season and therefore have a much earlier RTS date than their recorded RTS date. This is a limitation of injury reporting in the NFL that can lead to exaggerated days lost to injury. While ideally it would be more accurate to determine their ‘healed’ date over the offseason, given the NFL reporting system this is unobtainable. Analyzing the MOI was also limited to the number of available footages for the injuries as merely 38 injuries were available for footage review out of 464 injuries reported in the IR list. Although the entire game footage was reviewed for each injury, a majority of these did not include adequate camera angles of the injury itself. This flaw is thus susceptible to selection bias as it applies the MOIs sustained in 8% of the injuries and assumes it applies to all 464 injuries. Of note, the roster used on the league’s official website did not explicitly state whether an injury happened during practice or competition thus there could be confounding factors in injuries during competition versus during practice. Despite these limitations, to our knowledge, this epidemiological study provides the most comprehensive overview of neck injury in the NFL to date. The strength of this study was the ability to define an incidence rate of overall neck injuries based on the number of team plays as well as to find the positions with the highest risk of neck injuries.

## Conclusion

There is a high incidence of neck injuries among NFL athletes with predictable MOIs including head-to-head contact, head-down tackling, direct extremity-to-head contact, and head-to-ground contact. Defensive players were more likely to sustain neck injuries compared to offensive players. Defensive back was the most common position to sustain a neck injury.

## Data Availability

The datasets generated and/or analyzed during the current study are available in the NFL repository, https://www.nfl.com/injuries, https://www.nfl.com/stats/team-stats.
